# Development of clinical competence – a longitudinal survey of nurse practitioner students

**DOI:** 10.1186/s12912-021-00627-x

**Published:** 2021-07-16

**Authors:** I. Taylor, P. C. Bing-Jonsson, E. Finnbakk, S. Wangensteen, L. Sandvik, L. Fagerström

**Affiliations:** 1 Faculty of Health and Social Sciences, University of South-Eastern Norway (USN), P. O. Box 235, 3603 Kongsberg, Norway; 2 Lovisenberg Diaconal University College, Oslo, Norway; 3 Faculty of Education and Welfare Studies, Åbo Akademi University, Vaasa, Finland; 4 Faculty of Medicine and Health Science in Gjøvik, Norwegian University of Science and Technology (NTNU), Gjøvik, Norway

**Keywords:** Advanced practice nursing, Nurse practitioner, Clinical competence, Development, Self-assessment, Nurse education

## Abstract

**Background:**

In order to achieve a sustainable standard of advanced clinical competence for nurse practitioners leading to a credible role, it is important to investigate the development of clinical competence among nurse practitioner students.

**Aim:**

The aim of the present study is to analyse the development of nurse practitioner students’ self-assessed clinical competence from the beginning of their education to after completion of their clinical studies.

**Design:**

The study involved the application of a longitudinal survey design adhering to STROBE guidelines.

**Methods:**

The participants consisted of 36 registered nurses from a nurse practitioner programme at a Norwegian university. The Professional Nurse Self-Assessment Scale II was used for data collection during the period August 2015 to May 2020.

**Results:**

The students developed their clinical competence the most for direct clinical practice. Our findings are inconclusive in terms of whether the students developed clinical competence regarding consultation, coaching and guidance, and collaboration. However, they do indicate a lack of development in some aspects of clinical leadership. The students with the lowest level of clinical competence developed their clinical competence regarding direct clinical practice significantly more than the students with the highest level of clinical competence. The differences between students with high and low levels of clinical competence were levelled out during their education. Thus, the students as a whole became a more homogenous group after completion of their clinical studies. Previous work experience in primary healthcare was a statistically significant, yet minor, predictor of the development of clinical competence.

**Conclusion:**

Our findings indicate that the students developed their clinical competence for direct clinical practice in accordance with the intended learning outcomes of the university’s Master’s programme and international standards for nurse practitioners. It is imperative that the clinical field supports nurse practitioners by facilitating extended work-task fits that are appropriate to their newly developed clinical competence. We refrain from concluding with a recommendation that prior clinical work experience should be an entry requirement for nurse practitioner programmes. However, we recommend an evaluation of the nurse practitioner education programme with the aim of investigating whether the curriculum meets the academic standards of clinical leadership expected in advanced level of nursing.

## Background

Advanced practice nursing (APN) has been introduced as a means of delivering high quality, safe and affordable healthcare [1]. At least one-third of all OECD (Organisation for Economic Cooperation and Development) countries reported in 2012–2013 that during the previous 5 years, they had expanded their scopes of practice of non-physicians, including advanced roles for nurses [2]. Research indicates that APN is beneficial in terms of achieving health outcomes for patients and patient satisfaction [3–5]. On 1 February 2020, Norway introduced regulations for registered nurses (RNs) with a Master’s degree in APN [6] as part of an effort to meet the challenges facing the primary health care sector [7].

According to International Council of Nurses (ICN) guidelines [1], an APN is an RN: ‘with the expert knowledge base, complex decision-making skills and clinical competencies for Advanced Nursing Practice’ ([1] p. 4) who holds a minimum of a Master’s degree. Clinical nurse specialist (CNS) and nurse practitioner (NP) are usually the two most recognised APN roles [1]. The ICN [1] provides the following definition of an NP: ‘A Nurse Practitioner is an Advanced Practice Nurse who integrates clinical skills associated with nursing and medicine in order to assess, diagnose and manage patients in primary healthcare (PHC) settings and acute care populations as well as ongoing care for populations with chronic illness’ ([1] p. 4).

An NP is an autonomous clinician with a clinical expertise that combines the diagnosis and treatment of health conditions and the prescription of medication based on evidence-informed guidelines and nursing principles. Moreover, NPs emphasise disease prevention and health management in their practice [1]. Research demonstrates that patients receiving care from NPs experience fewer unnecessary emergency room visits, reduced waiting times, and fewer hospital admissions and readmissions [8–10]. National contexts and the regulatory policies within which NPs practice determine their levels of autonomy and accountability [1]. Norway is presently in its first phase of implementing the NP role, and NPs do not currently have prescribing rights in Norway.

In order to meet the growing need for clinical competence in the care of an increasingly ageing population, as well as patients with complex health conditions, one university in Norway is currently offering a part-time (three- to- four-year) Master’s degree programme for NPs with a 120 European Credit Transfer System (ECTS). The programme was launched in 2015 and includes courses in advanced health and physical assessment, advanced pathophysiology and advanced pharmacology. It also offers three courses focusing on elective specialization in geriatric assessment, acute medical assessment and specialization in wound care and acute pain treatment. All NP students in the programme are expected to submit a Master’s thesis. The programme is consistent with both the ICN [1] definition of an NP and Fagerström’s [11] Nordic APN model, which incorporates the nurse-patient relationship, eight core competencies and critical contextual factors.

The definition of clinical competence within nursing has been the subject of much debate, but there seems to be an international consensus that the concept must be viewed from a holistic perspective [12] that includes the application of complex combinations of knowledge, performance, skills, values and attitudes [13]. This holistic view appears to be in line with the complementary view of knowledge that underpins the Nordic APN model, representing a synthesis of the three Aristotelian dimensions of knowledge: scientific knowledge (*episteme*), expertise (*technê*), and practical wisdom (*phronêsis*) [14]. An APN nurse’s clinical competence can be described as the result of a process in which these three dimensions of knowledge are synthesized to become ‘knowledge in action’ [11].

In order to investigate the clinical competence and the need for further training of NP/APN students, two studies have been carried out using a questionnaire called the Professional Nurse Self-Assessment Scale of clinical core competencies II (PROFFNurseSAS II), which is grounded in the Nordic APN model [15]. The sample included in the first study involved specialist and APN students from Iceland, the Netherlands, Sweden, Norway and the United Kingdom, surveyed on completion of their education [15]. The sample included in the second study involved students from three different NP/APN education programmes in Norway, surveyed at the beginning of their education [16]. Both studies found that the students self-assessed their clinical competence to be highest in aspects related to taking responsibility and their need for further training with regard to knowledge of medication [15, 16].

The ICN [1] emphasises that the educational preparation that enables students to meet the qualifications for NP practice is crucial to the credibility and sustainability of the NP concept. Since continuous evaluation is a critical contextual factor in the Nordic APN model [11], it is important to investigate the development NP students’ clinical competence in a Norwegian context. Moreover, it is especially important for countries like Norway, in which the NP/APN role is at an early stage of its development, to evaluate whether it is evolving according to international standards. Previous empirical research on clinical competence within nursing has been dominated by cross-sectional design studies, so we recognise a need for research employing a longitudinal design that track changes in clinical competence throughout a nurse’s education [17]. Thus, with the aim of exploring the development of nursing students’ clinical competence during an NP Master’s programme, the present work describes and analyses their self-assessed clinical competence using a longitudinal design study facilitated by the aforementioned PROFFNurse SAS II questionnaire.

## Methods

### Aim

The aim of the present study is to analyse the development of NP students’ self-assessed clinical competence from the beginning of their education (baseline) to after completion of their clinical studies (follow-up).

The following research questions regarding the students’ education were addressed:

- What were the most and least developed items from baseline to follow-up?
- How did the lowest self-assessed items at baseline develop for students with high and low clinical competence?
- Are *clinical work experience* and *previous higher education* predictors for the development of clinical competence?

### Design

A longitudinal survey design is applied in this study, and the ProffNurseSAS II questionnaire employed for the NP students’ to self-assess their clinical competence at baseline and follow up.

### Questionnaire

The PROFFNurseSAS II questionnaire used in this study aims to measure the clinical competence of nurses at different educational levels from a holistic and lifelong learning perspective [15]. The questionnaire builds on the validated questionnaire PROFFNurseSAS I [18], which consisted of six components: direct clinical practice, professional development, ethical decision-making, clinical leadership, cooperation and consultation, and critical thinking. Wangensteen [15] sought to improve the questionnaire and developed a modified version containing 50 items subdivided along two scales: the *A-scale* for self-assessed clinical competence, and the *B-scale* for self-assessed need for further training [15].

The PROFFNurseSAS II questionnaire has been evaluated for content validity [15] and reliability (the Cronbach’s alpha value for the A-scale was 0.936) [16]. Since the aim of the present study addresses the development of clinical competence, it reports findings on the A-scale.

### Participants

The survey involved the recruitment of a sample of RNs attending an NP programme at a Norwegian university. The first inclusion criterion was enrolment as a first-year student in the programme during their first semester. The second criterion centred on the students’ completion of their clinical studies (450 h) and the passing of their Objective Structured Clinical Assessment (OSCA). A total of 46 NP students from four cohorts that met the inclusion criteria were invited to participate. Among these, 36 were included in the present study, a response rate of 78%.

A power analysis was performed to evaluate sample size. The standard deviations of the total A-score from the first and second investigations were 0.90 and 0.74, respectively. It is possible, given that there were 36 APN students in the follow-up investigation, that the test power will be at least 90% if the mean difference in the total A-score between the first and second estimations is at least 0.50. Thus, we are satisfied that 36 students appears to be an appropriate sample size for the present study.

### Data collection

The students completed the questionnaire twice: initially at the beginning of their NP education (baseline), and then later after they had completed their clinical studies (follow-up). The interval between surveys was approximately 2 years. The NP education is a part-time programme, in which all the students are assigned an individual study plan stating when and where their clinical studies are conducted, according to their needs and wishes. This provides the students with some flexibility, enabling some to spend more time completing their clinical studies than others. For this reason, there are slight differences between the two measuring points, although an approximate 2-year period applied to all students.

Data were collected using the questionnaire PROFFNurseSAS II, which includes 50 items with responses ranged on a scale from 1 to 10, where 1 indicates a poor, and 10 an excellent, level of clinical competence. The questionnaire also includes an option for scoring ‘entirely missing competence’, which is quantified as zero. The questionnaire also includes the option ‘competency not covered in the programme’, which was treated as an invalid value and not included in the analysis. The number of students who selected this option is set out in the Tables. A number of sociodemographic variables were also collected, including gender, age, years of clinical work experience as an RN, area of work experience (i.e., as a specialist or in primary health care), and previous higher education qualifications above a Bachelor’s degree in nursing, as recognised by the ECTS.

The students were first invited to participate in the baseline investigation by means of a printed handout of the questionnaire distributed during an NP programme lecture. As part of the second invitation, to the follow-up investigation, the students were given the opportunity to respond to the questionnaire online. Data collection was carried out between August 2015 and May 2020 by two of the present authors (IT and LF).

### Ethical considerations

The project was approved by the Norwegian Centre for Research Data (NSD: approval no. 52648). Information was provided to the informants both at baseline (orally and on a written handout) and follow-up (via e-mail). The students were also informed about participant anonymity and their right to withdraw from the study at any time, without giving any reason. An ethical issue arises in situations where the nurse educators are also researchers conducting the study, and their students are participants. Such students may feel under some duress for fear that non-participation will impact on their progress or learning experience within the programme [19]. In order to address this concern, a PhD student with no teaching affiliation to the faculty (IT) assumed responsibility for collecting all the data, except in the case of one of the cohorts for which a former education programme leader (LF) collected data at baseline. It was emphasised to all the students that their responses would not affect their teachers’ evaluations or their examination grades. A scrambling key was created that enabled directly identifiable information to be stored separately from the data. Only the first author (IT) had access to the scrambling key.

### Data analysis

The software IBM SPSS® Statistics 26.0 for Windows was used for data analysis. Of the total number of participants, 79% (*n* = 31) responded to all 50 items in the questionnaire. The baseline and follow-up investigations had total response rates of 85% (*n* = 33) and 87% (*n* = 34), respectively. Only participants with fewer than 10 missing items (18% of the total number of items) were included in the study. This criterion resulted in the exclusion of three participants. Thus, among the 39 NP students who responded to the questionnaire (a response rate of 80%), 36 were included in the data analysis. The case mean substitution technique is recommended in self-assessment studies [20] and it was thus used to replace missing data for participants who met the inclusion criteria (*n* = 5).

The data were expressed as frequencies, percentages and means to summarize the students’ demographical variables, items and total mean. Paired sample t-tests (two tailed) were conducted on participants’ total mean scores and individual items during comparisons of baseline and follow-up scores. An independent sample t-test was used when comparing NP students with high and low self-assessed clinical competencies.

Multiple linear regression analyses were conducted in situations where the total mean score for self-assessed clinical competence at follow-up was used as a dependent variable. The independent variables were as follows; (a) total mean score for the students’ self-assessed clinical competence at baseline, (b) age, (c) years of clinical work experience as an RN (overall, in primary health care and specialist health care, respectively) and (d) previous level of higher education above a Bachelor’s degree in nursing (measured in ECTS credits). Forced entry was used for all the predicting variables [21], which means that all independent variables were entered simultaneously. A backward variable selection method was subsequently applied by which predictors were removed one by one when they were not statistically significant, until all the remaining predictors were recognised as significant. The aim of this process was to build a model containing only statistically significant predictors [21]. Follow-up scores were adjusted for the baseline when linear regressions were performed, as recommended by Vickers and Altman [22],

The assumptions underlying the t-tests and linear analyses were checked and found to be adequately met, and *p* values below 0.05 were considered statistically significant.

It is important in this study to recognize a potential ceiling effect resulting from selection of the highest value at baseline, which may lead to an erroneous conclusion of no improvement [23]. In the present study, a ceiling effect was considered to be present at baseline if more than 15% of the participants self-assessed themselves with the highest possible score.

## Results

The participating students (*n* = 36) consisted of 33 females and three males, with a mean age of 41 (range: 26–59). The mean number of years with clinical work experience as an RN was 13.0 (range: 4–33). All the participants were part-time students. Their mean number of years of clinical work experience in primary health care was 7.3 (range: 0–27), and 4.7 (range: 0–17) in specialist health care. Before entering their NP programmes, 23 students (64%) had previous educational qualifications above the level of a Bachelor’s degree in nursing. Among these, 18 students (50%) had obtained more than 30 ECTS credits.

The total mean score of clinical competence at baseline was 6.83 (SD = 0.90, range: 4.94–8.64), and 8.22 (SD = 0.74, range: 6.68–9.77) at follow-up. The mean difference between baseline and follow-up was 1.39 (SD = 0.80; range: − 0.68–2.84; 95% CI: − 1.58–1.04; *p* < 0.001). Among the 50 items in the questionnaire, 44 increased significantly (not shown in table).

The ten items that increased most between baseline and follow-up are presented in Table 1. Eight of these related to direct clinical practice, such as history-taking, physical examination, differential diagnosing and medication, while two were related to health promotion and illness prevention, and support and guidance provided to the patient. These items increased from 1.66–2.80 between baseline and follow-up, and all items increased significantly.

**Table 1:** Top 10 developed self-assessed items between baseline and follow-up (paired sample t-test)

Item no.	Item	Baseline Mean (SD)	Follow-up Mean (SD)	Difference Mean
**14**	**I systematically gather information from each patient about her/his health resources**	**5.49 (1.93)**	**8.29 (1.05)** ^a^	**2.80**^⁎⁎^
**8**	**I interpret, analyse and reach alternative conclusions about patients’ health conditions after a detailed mapping of health history and health assessment (physical examination)**	**5.31 (1.86)**	**7.75 (1.27)**	**2.44**^⁎⁎^
**15**	**I have knowledge of the interactions of various types of medication and what side-effects they may cause for the patients I am responsible for**	**5.97 (2.09)**	**8.25 (1.03)**	**2.28**^⁎⁎^
**13**	**I develop and administer health-promoting and illness-preventive actions for patients**	**5.89 (2.00)**	**8.11 (1.02)** ^a^	**2.22**^⁎⁎^
**9**	**I apply both subjective and objective methods when examining, treating and caring for patients**	**6.14 (1.79)**	**8.33 (1.22)**	**2.19**^⁎⁎^
**7**	**I exclude differential diagnoses when assessing patients’ health conditions**	**5.61 (1.93)**	**7.72 (1.56)**	**2.11**^⁎⁎^
**1**	**I am independently responsible for health assessment (systematic physical examination), examinations and treatment of patients with complicated medical conditions**	**5.36 (1.71)**	**7.31 (1.77)**	**1.95**^⁎⁎^
**2**	**I am independently responsible for health assessment (systematic physical examination), examinations and treatment of patients with uncomplicated medical conditions**	**6.36 (1.82)**	**8.28 (1.06)**	**1.92**^⁎⁎^
**47**	**I give health promotion and illness preventive recommendations in accordance with national guidelines to patients**	**5.83 (2.33)**	**7.49 (2.05)** ^a^	**1.66**^⁎⁎^
**27**	**I support and guide patients in mastering their illnesses and health problems**	**5.83 (2.33)**	**7.49 (2.05)**	**1.66**^⁎⁎^

*Note:* Bold font indicates statistically significant differences

^a^Competency not covered in the programme: item no. 13—1 student; item no. 14—1 student; and item no. 47—1 student

^⁎⁎^p < 0.001

The ten items that increased least between baseline and follow-up are presented in Table 2. These related to fragmented aspects of clinical competence such as responsibility, cooperation with the physician, decision-making, improvements in the workplace, and the use of electronic devices such as telephones and e-mail when assessing the patient. These items increased from 0.12–0.75 between baseline and follow-up, and five items increased significantly. A ceiling effect was observed for seven items.

**Table 2:** Lowest 10 developed self-assessed items between baseline and follow-up (paired sample t-test)

Item no.	Item	Baseline Mean (SD)	Follow-up Mean (SD)	Difference Mean	Ceiling effect observed
35	I experience a division of responsibility between the physician and me as a nurse	7.44 (1.80)	7.56 (1.99)	0.12	Yes
19	I improve routines/systems that fail to meet the needs of patients at my workplace	6.60 (2.15)	6.89 (2.06) ^a^	0.29	No
46	I give health promotion advice and recommendations to patients by telephone, e-mail or other electronic devices	4.71 (2.46)	5.07 (3.42) ^a^	0.36	No
50	I report all incidents in accordance with the actual patient safety system	7.14 (2.14)	7.58 (2.03)	0.44	Yes
29	I take active responsibility for creating a good working environment	8.06 (1.49)	8.53 (1.11)	0.47	Yes
**31**	**I make my own decisions in my work**	**7.89 (1.60)**	**8.36 (1.76)**	**0.47**^⁎^	No
**36**	**I cooperate well with the physician**	**8.53 (1.23)**	**9.03 (1.13)**	**0.50**^⁎^	Yes
**39**	**I am cognisant of when my medical knowledge is insufficient when assessing patients’ health conditions**	**8.61 (1.32)**	**9.11 (0.89)**	**0.50**^⁎^	Yes
**32**	**I take full responsibility for my own actions**	**8.67 (1.33)**	**9.33 (0.96)**	**0.66**^⁎^	Yes
**20**	**I am actively responsible for my own professional development**	**8.28 (1.70)**	**9.03 (0.88)**	**0.75**^⁎^	Yes

*Note:* Bold font indicates statistically significant differences

^a^Competency not covered in the programme: item no. 19—1 student; and item no. 46—8 students

^⁎^*p* < 0.05

The 10 items ranked lowest at baseline are presented in Table 3. These related to direct clinical practice, improvement in the workplace, health promotion and illness prevention, and the use of electronic devices such as telephones and e-mail when assessing the patient. The items increased from 0.36–2.8 between baseline and follow-up, and eight items increased significantly.

**Table 3:** 10 lowest self-assessed items at baseline with follow-up scores and difference (paired sample t-test)

Item no.	Item	Baseline Mean (SD)	Follow-up Mean (SD)	Difference Mean
45	I assess patients’ health needs by telephone, e-mail or other electronic devices	4.70 (2.32)	5.96 (2.91)^a^	1.26
46	I give health promotion advice and recommendations to patients by telephone, e-mail or other electronic devices	4.71 (2.46)	5.07 (3.42) ^a^	0.36
**8**	**I interpret, analyse and reach alternative conclusions about patients’ health conditions after a detailed mapping of health history and health assessment (physical examination)**	**5.31 (1.86)**	**7.75 (1.27**	**2.44**^⁎^
**1**	**I am independently responsible for health assessment (systematic physical examination), examinations and treatment of patients with complicated medical conditions**	**5.36 (1.71)**	**7.31 (1.77)**	**1.95**^⁎⁎^
**18**	**I take responsibility for competence development at my workplace**	**5.49 (2.55)**	**7.06 (2.51)**	**1.57**^⁎⁎^
**14**	**I systematically gather information from each patient about her/his health resources**	**5.49 (1.93)**	**8.29 (1.05)**	**2.80**^⁎⁎^
**17**	**I participate in quality development at my workplace**	**5.56 (2.55)**	**6.41 (2.69)**	**0.85**^⁎^
**7**	**I exclude differential diagnoses when assessing patients’ health conditions**	**5.61 (1.93)**	**7.72 (1.56)**	**2.11**^⁎⁎^
**6**	**I evaluate and modify patients’ medical treatment**	**5.74 (1.74)**	**7.06 (1.47)** ^a^	**1.32**^⁎⁎^
**47**	**I give health promotion and illness preventive recommendations in accordance with national guidelines to patients**	**5.83 (2.33)**	**7.49 (2.05)**	**1.66**^⁎⁎^

*Note:* Bold font indicates statistically significant differences

^a^Competency not covered in the programme: item no. 6—1 student; item no. 18—1 student; item no. 45—9 students; and item no. 46—8 students

^⁎^p < 0.05

^⁎⁎^p < 0.001

We extracted two groups from the total sample. One group represents the third of the students with the highest total mean for clinical competence (*n* = 12; total mean: 7.93; SD: 0.49; range: 7.31–8.75), and the other group represents the third of the students with the lowest total mean for clinical competence (n = 12; total mean: 5.91; SD: 0.46; range: 4.98–6.42). In the group with high clinical competence, the 10 lowest self-assessed items at baseline are presented in Table 4. In this group, eight items increased significantly. In the group with low clinical competence, the 10 lowest self-assessed items at baseline are presented in Table 5. In this group, eight items increased significantly. When the 10 lowest self-assessed items at baseline for students with high and low clinical competence (Tables 4 and 5) were compared, 8 items were found in both groups (items in grey in Tables 4 and 5). Among these eight items, five concerned direct clinical practice, two concerned assessing the patient with electronic devices and one concerned competence development at the workplace. Among the 50 items, 22 items increased significantly more for students with low clinical competence than for students with high clinical competence (Table 6). The ceiling effect was observed for 14 items, but this did not include the items concerning direct clinical practice.

**Table 4:** Students with higher clinical competence and the 10 lowest self-assessed items at baseline (paired sample t-test)

Item no.	Item	Baseline Mean (SD)	Follow-up Mean (SD)	Difference Mean
45	I assess patients’ health needs by telephone, e-mail or other electronic devices	4.50 (2.39)	5.75 (3.06)^a^	1.25
46	I give health promotion advice and recommendations to patients by telephone, e-mail or other electronic devices	5.56 (2.79)	5.78 (3.87 ^a^	0.22
**8**	**I interpret, analyse and reach alternative conclusions about patients’ health conditions after a detailed mapping of health history and health assessment (physical examination)**	**5.75 (1.91)**	**8.25 (1.36)**	**2.50**^⁎⁎^
**1**	**I am independently responsible for health assessment (systematic physical examination), examinations and treatment of patients with complicated medical conditions**	**5.92 (1.88)**	**7.67 (2.23)**	**1.75**^⁎^
**18**	**I take responsibility for competence development at my workplace**	**6.17 (2.37)**	**7.92 (1.62)**	**1.75**^⁎^
**7**	**I exclude differential diagnoses when assessing patients’ health conditions**	**6.58 (1.68)**	**8.42 (1.00)**	**1.84**^⁎^
**14**	**I systematically gather information from each patient about her/his health resources**	**6.73 (1.85)**	**8.64 (1.29)** ^a^	**1.91**^⁎^
6	I evaluate and modify patients’ medical treatment	6.67 (1.56)	7.42 (1.68)	0.75
**15**	**I have knowledge of the interactions of various types of medication and what side-effects they may cause for the patients I am responsible for**	**6.67 (1.83)**	**8.75 (0.97)**	**2.08**^⁎^
**13**	**I develop and administer health-promoting and illness-preventive actions for patients**	**7.00 (1.35)**	**8.67 (0.89)**	**1.67**^⁎^

*Notes:* Bold font indicates statistically significant differences

^a^ Competency not covered in the programme: item no. 14—1 student; item no. 45—4 students; and item no. 46—3 students

^⁎^ p < 0.05

**Table 5:** Students with lower clinical competence and the 10 lowest self-assessed items at baseline (paired sample t-test)

Item no.	Item	Baseline Mean (SD)	Follow-up Mean (SD)	Difference Mean
17	I participate in quality development at my workplace	3.45 (2.21)	4.82 (3.28)^a^	1.37
16	I generate a creative learning environment for staff at my workplace	3.58 (1.83)	5.92 (1.88)	2.34
**18**	**I take responsibility for competence development at my workplace**	**3.82 (1.94)**	**5.55 (3.01)**^a^	**1.73**^⁎^
**13**	**I develop and administer health-promoting and illness-preventive actions for patients**	**4.09 (2.12)**	**7.73 (1.27)**^a^	**3.64**^⁎⁎^
46	I give health promotion advice and recommendations to patients by telephone, e-mail or other electronic devices	4.20 (2.20)	4.50 (3.54)^a^	0.30
**15**	**I have knowledge of the interactions of various types of medication and what side-effects they may cause for the patients I am responsible for**	**4.25 (2.14)**	**7.75 (0.97)**	**3.50**^⁎⁎^
**14**	**I systematically gather information from each patient about her/his health resources**	**4.25 (1.55)**	**8.08 (1.00)**	**3.83**^⁎⁎^
45	I assess patients’ health needs by telephone, e-mail or other electronic devices	4.44 (2.01)	5.44 (3.43)^a^	1.00
**8**	**I interpret, analyse and reach alternative conclusions about patients’ health conditions after a detailed mapping of health history and health assessment (physical examination)**	**4.42 (1.51)**	**7.50 (1.24)**	**3.08**^⁎⁎^
**7**	**I exclude differential diagnoses when assessing patients’ health conditions**	**4.42 (1.83)**	**7.50 (1.17)**	**3.08**^⁎⁎^

*Notes:* Bold font indicates statistically significant differences

^a^Competency not covered in the programme: item no. 13—1 student; item no. 17—1 student; item no. 45—3 student, and item no. 46—2 students

^⁎⁎^p < 0.001

**Table 6:** Students with high clinical competence vs low clinical competence (independent sample t-test)

Item no.	Item	Group	Baseline Mean (SD)	Follow-up Mean (SD)	Difference Mean	Ceiling effect
2	I am independently responsible for health assessment (systematic physical examination), examinations and treatment of patients with uncomplicated medical conditions	High clinical competence	7.75 (1.71)	8.50 (1.09)	0.75^⁎^	No
		Low clinical competence	5.42 (1.68)	8.17 (0.94)	2.75^⁎^	
3	I plan and prioritise nursing and medical interventions	High clinical competence	7.75 (0.75)	8.58 (1.08)	0.83^⁎^	No
		Low clinical competence	5.58 (1.51)	8.00 (1.35)	2.42^⁎^	
4	I identify patients’ health problems	High clinical competence	7.92 (1.17)	8.67 (1.16)	0.75^⁎^	No
		Low clinical competence	6.42 (1.51)	8.33 (0.99)	1.91^⁎^	
9	I apply both subjective and objective methods when examining, treating and caring for patients	High clinical competence	7.75 (0.87)	8.92 (1.17)	1.17^⁎^	No
		Low clinical competence	5.25 (1.36)	8.08 (1.08)	2.83^⁎^	
12	I identify changes in patients’ health and medical conditions	High clinical competence	7.92 (1.17)	8.75 (0.87)	0.83^⁎^	No
		Low clinical competence	5.58 (2.35)	8.67 (0.65)	3.09^⁎^	
13	I develop and administer health-promoting and illness-preventive actions for patients	High clinical competence	7.00 (1.35)	8.67 (0.89)	1.67^⁎^	No
		Low clinical competence	4.09 (2.12)	7.73 (1.27) ^a^	3.64^⁎^	
14	I systematically gather information from each patient about her/his health resources	High clinical competence	6.73 (1.85)	8.64 (1.29) ^a^	1.91^⁎^	No
		Low clinical competence	4.25 (1.55)	8.08 (1.00)	3.83^⁎^	
20	I am actively responsible for my own professional development	High clinical competence	9.33 (0.89)	9.50 (0.80)	0.17^⁎^	Yes
		Low clinical competence	7.17 (2.04)	8.92 (0.90)	1.75^⁎^	
21	I take patients’ mental health needs (mood swings, feelings of hopelessness, depression, etc.) into account when assessing and planning for the health and life situation of patients	High clinical competence	8.33 (1.07)	8.75 (1.01)	0.42^⁎^	Yes
		Low clinical competence	6.42 (1.62)	8.25 (1.06)	1.83^⁎^	
22	I take patients’ spiritual health needs (feelings of meaninglessness, existential needs, beliefs, fear of death, etc.) into account when assessing and planning for the health and life situation of patients	High clinical competence	8.08 (1.56)	8.00 (1.54)	−0.08^⁎^	Yes
		Low clinical competence	5.67 (1.83)	7.83 (1.03)	2.16^⁎^	
23	I take patients’ physical health needs (illness, pain, disabilities, etc.) into account when assessing and planning for the health and life situation of patients	High clinical competence	8.92 (0.90)	9.08 (0.79)	0.16^⁎⁎^	Yes
		Low clinical competence	6.75 (1.14)	8.58 (0.79)	1.83^⁎⁎^	
24	I act ethically when caring for patients	High clinical competence	8.83 (0.84)	9.17 (0.84)	0.33^⁎^	Yes
		Low clinical competence	6.83 (1.80)	8.83 (0.72)	2.00^⁎^	
25	I identify and assume responsibility for patients’ own health resources in planning nursing care	High clinical competence	8.08 (1.44)	8.33 (1.07)	0.25^⁎⁎^	Yes
		Low clinical competence	5.33 (1.72)	8.42 (1.00)	3.08^⁎⁎^	
26	I take patients’ social health needs (leisure activities, friends, financial situation, etc.) into account when assessing and planning for the health and life situation of patients	High clinical competence	7.55 (1.44)	8.18 (1.33) ^a^	0.63^⁎^	No
		Low clinical competence	4.58 (1.93)	7.42 (1.17)	2.83^⁎^	
27	I support and guide patients in mastering their illnesses and health problems	High clinical competence	8.50 (1.09)	8.42 (1.08)	−0.08^⁎⁎^2.67^⁎⁎^	Yes
		Low clinical competence	5.50 (1.83)	8.17 (0.72)		
30	I put emphasis on patients’ own wishes when assessing and planning for nursing care and medical treatment	High clinical competence	8.50 (1.73)	9.00 (0.85)	0.50^⁎^	Yes
		Low clinical competence	6.08 (2.11)	8.50 (1.00)	2.42^⁎^	
32	I take full responsibility for my own actions	High clinical competence	9.17 (0.94)	9.25 (1.14)	0.08^⁎^	Yes
		Low clinical competence	8.00 (1.71)	9.25 (1.06)	1.25^⁎^	
34	I understand the consequences my decisions may have for patients	High clinical competence	8.67 (1.16)	9.17 (0.94)	0.5^⁎^	Yes
		Low clinical competence	7.17 (1.47)	9.00 (0.95)	1.83^⁎^	
37	I consult other professional experts when required	High clinical competence	9.42 (0.79)	9.25 (0.97)	−0.17^⁎^	Yes
		Low clinical competence	7.17 (2.25)	9.00 (0.96)	1.83^⁎^	
38	I cooperate actively with other health professionals when coordinating patients’ nursing, care and treatment	High clinical competence	9.25 (0.87)	9.08 (0.90)	−0.17^⁎^	Yes
		Low clinical competence	7.33 (2.02)	9.00 (1.04)	1.67^⁎^	
39	I am cognisant of when my medical knowledge is insufficient when assessing patients’ health conditions	High clinical competence	9.58 (0.67)	9.25 (0.75)	−0.33^⁎^	Yes
		Low clinical competence	8.17 (1.47)	9.17 (0.58)	1.00^⁎^	
41	I reflect on my actions	High clinical competence	9.00 (0.74)	9.08 (1.08)	0.08^⁎^	Yes
		Low clinical competence	7.25 (2.09)	9.00 (0.74)	1.75^⁎^	

*Notes:* ^a^Competency not covered in the programme: item no. 13—1 student; item no. 14—1 student; item no. 26—1 student

^⁎^p < 0.05

^⁎⁎^p < 0.001

Previous clinical work experience as an RN within primary health care was found to be a statistically significant, yet minor, predictor for total mean clinical competence scores at follow-up, when adjusted for clinical competence at baseline (Table 7). This predictor variable explained 36.9% of the variance. Students with 10 years of previous clinical work experience in primary health care had a 0.35-point higher score for total clinical competence than students without. Neither work experience as an RN overall, work experience within specialist health care or previous education, were significant predictors.

**Table 7:** Regression: Self-assessment total mean at follow-up versus years of clinical work experience as an RN in primary health care, adjusted for total mean at baseline

Item	*B*	adjusted *R*^*2*^	*p*
Total mean baseline	= 0.401	= 0.369	= 0.003
Clinical work experience as an RN: Primary health care	= 0.035		= 0.038

## Discussion

### Clinical competence that developed the most

The aim of the present study is to analyse the development of NP students’ self-assessed clinical competence from the beginning of their education (baseline) to after completion of their clinical studies (follow-up). Among the 50 items in the PROFFNurseSAS II questionnaire, 45 increased significantly. The total clinical competence score increased by 1.39 points, resulting in a total score of 8.22 (of a maximum of 10) after the students had completed their clinical studies. This finding is consistent with that of Wangensteen [15], who found that APN students self-assessed their clinical competence at 8.08 on completing their education.

We found that, among the 10 items that increased the most between the beginning of the students’ NP education and following the completion of their clinical studies, 8 items concerned the direct clinical practice of history-taking, physical examination, differential diagnosis and medication. These items refer to medically-related clinical skills that highly proficient RNs must acquire in order to become NPs [1, 24]. This finding is in line with previous research, which has shown that direct clinical practice was what NP/APN students most desired to learn [15, 16], and what NP students perceived as the most important aspect of their clinical competence [25].

Clinical decision-making is an important aspect of an NP’s clinical competence. Tiffen et al. ([26] p.400) developed the following definition: ‘Clinical decision-making is a contextual, continuous, and evolving process, where data are gathered, interpreted, and evaluated in order to select an evidence-based choice of action’. Taylor, Bing-Jonsson, Johansen, Levy-Malmberg, and Fagerström [27] found that while NP students demonstrated some structured history-taking and physical assessment techniques, they struggled to demonstrate decision-making in their OSCE (first clinical exam) for a pre-clinical course that they were required to pass prior to starting their clinical studies. In the present study, all the participating students had completed their clinical studies. They had also passed their OSCA (a second clinical exam), during which they were asked to assess clinical preceptor-selected patients who had given their consent. Item no. 8 in the questionnaire – ‘I interpret, analyse and reach alternative conclusions about patients’ health conditions after a detailed mapping of health history and health assessment (physical examination)’ – is consistent with the aforementioned definition of clinical decision-making, and was ranked second among the items that increased its scores the most in the follow-up part of the present study. This skill is specific and essential to NPs, as is clearly stated in the APN Nordic model [11].

Previous research has shown that medication (interactions and side-effects) represented the item about which NP/APN students most wanted to learn [15, 16]. The present study found that this item was ranked third among the items in the questionnaire that increased its scores the most. A study has shown that RNs working in nursing homes are in need of a deeper understanding of the inter-complexity of age-specific diseases and medication in order to ensure that their clinical judgments and actions are appropriate and safe [28]. Another study revealed that among 243 graduating nursing students and 203 RNs who took a multiple choice test, 25% of the answers to questions related to medicines management indicated a high risk of error [29]. We thus regard it as a promising finding of the present study that the item related to medication was ranked third among those that increased its scores the most. Despite the fact that NPs in Norway have yet to be granted prescription rights, our NP programme course in pharmacology is extensive and compares well with those offered in countries where NPs are granted such rights. The findings in this study indicate that the students had developed their clinical competence for direct clinical practice in line with the ICN’s [1] NP definition and the intended learning outcomes of the NP Master’s programme, specifically in relation to advanced health and physical assessment, advanced pathophysiology and advanced pharmacology.

### Clinical competence that developed the least development

We found that the 10 items that increased the least between the start of students’ NP education and after the completion of their clinical studies concerned fragmented aspects of clinical competence such as responsibility, cooperation with the physician, decision-making, improvements in the workplace, and the use of electronic devices such as telephones and e-mail when assessing the patient. All of these items coincided with similarly fragmented aspects of clinical competencies set out in the Nordic APN-model, i.e., consultation, coaching and guidance, collaboration and leadership [11].

In the case of item 19 in the questionnaire (‘I improve routines/systems that fail to meet the needs of patients at my workplace’), the students reported average scores at the beginning of their education, and scores for this item did not increase significantly on completion of their clinical studies. This item is considered to be important as a measure of a student’s clinical competence in relation clinical leadership in the workplace. According to the ICN [1], NPs are clinical leaders who can influence health service delivery and the profession at large. Thus, an assessment of the development of students’ clinical competence at advanced nursing level must include an evaluation of their ability as clinical leaders. Indeed, leadership has been reported to be a key factor in an advanced practitioner’s ability to influence innovation, improve clinical practice and health care delivery, and advance the nursing/midwifery professions [30]. Moreover, the leadership aspect of the APN role is embedded in the statutory regulations governing APN Master’s programmes offered in Norway [31], which conform in most respects to national policy and competency standards relating to NP education in countries such as Australia, England, the United States and South Africa [32–35]. However, the finding in the present study may indicate that the NP students did not develop sufficiently with regard to clinical competence in some aspects of clinical leadership.

The students’ self-assessment of item no. 46—‘I give health promotion advice and recommendations to patients by telephone, e-mail or other electronic devices’—was the second-lowest at the beginning of their education. This item did not increase significantly on completion of their clinical studies. This outcome is in line with previous findings showing that NP/APN students consistently reported the lowest scores for this item [15], and were neutral in their attitudes as to whether this was an important factor in the further training [16]. We note in passing that experience from the Covid-19 pandemic has demonstrated that digital health care is more relevant today than it has ever been.

A ceiling effect was observed for seven of the ten items that increased their scores the least. Since a ceiling effect can be indicative of an incomplete scale [23], it may be argued that some of the items in the PROFFNurseSAS II questionnaire have limitations regarding their value as measures of clinical competence at an advanced level. However, a ceiling effect may reflect other than purely statistical issues [23] and NP students may have been reporting a maximum score for items relevant to their experiences as an RN. There are distinct differences between the levels of clinical competence among RNs and NPs. NPs have a broader degree of autonomy due to their advanced in-depth critical decision-making skills [1]. Due to the recent introduction of the NP role in Norway, many of the students in the present study did not associate with other NPs who could act as role models and indicators of what might be expected in terms of clinical competence at advanced NP level. Thus, in this respect, the present study has some limitations with regard to its measurement of some aspects of clinical competence at NP level.

### Students with high and low clinical competence

When selecting the ten items with the lowest mean scores at the beginning of the students’ education, the high- and low-clinical competence groups revealed surprising similarity. With the exception of two items, those selected were identical in both groups. Even more surprisingly, in the case of 22 items, students with a low clinical competence at baseline increased their scores significantly more than those starting with a high clinical competence. A ceiling effect was observed for 14 of the 22 items, but not for those relating to direct clinical practice, which represent the central competencies set out in the Nordic APN model [11]. This means that the students with low clinical competence increased their clinical competence of direct clinical practice more that the students with higher clinical competence. In short, the differences between students with high and low levels of clinical competence at baseline were levelled out during their education. As a consequence, the students evolved into a more homogenous group with similar levels of clinical competence in relation to direct clinical practice after the completion of their clinical studies.

It is important to implement set standards in NP education as a means of achieving consistency in the educational preparation and authorization of NPs [36]. However, there are both positive and negative aspects in having all students develop their clinical competence to the same level. Stensaker and Prøitz [37] have expressed the concern that our perception of quality in education is conflicted. On the one hand, educational programmes are broad-based and generalised in an attempt to promote inclusivity. Others, on the other hand, education policy evolve as narrow and elitist in pursuit of promoting excellence. A challenge currently facing NP education is the fact that programmes seek simultaneously to educate students to be safe practitioners at an advanced level [38] while also encouraging them to be highly autonomous pioneers with the ability to make a difference to the quality of patient care [39]. We argue that NP education should aim to level up differences in clinical competence in order to ensure that the NP role represents a sustainable standard that contributes to improvements in patient safety.

### Work experience and the development of clinical competence

Previous research has not found an association between the clinical competence and work experience of RNs in primary health care [40], or among RNs and critical care nurses working in intensive care units [41]. This is in line with the present authors’ previous research [16], during which we also did not establish work experience in primary health care as a significant predictor of clinical competence among APN/NP students at the beginning of their education. This finding is also supported by that of Wangensteen [15]. However, in the present study, we found that work experience in primary health care was a statistically significant, yet minor, predictor of the total mean score reported by students after they had completed their clinical studies, following adjustment for the total mean score reported at baseline. This could mean that, even though previous work experience was not associated with clinical competence at the beginning of their education, the students who had worked within primary health care utilized their work experience in a way that enabled them to develop clinical competence. Knowles, Holton, Swanson, and Robinson [42] emphasise that the richest resources for learning reside in the adult learners themselves, and recommend teaching techniques that tap in to the learners’ own experiences. Offering students the opportunity to draw on their prior work experience during their Master’s education has been shown to promote learning, especially when educators facilitate the students’ critical reflections on their experiences and provided feedback on their performance [43]. Students who participated in the present study were specifically encouraged to apply their previous work experience during the programme’s coursework and lectures. Our findings may therefore indicate that the NP Master’s programme is well suited to students who have prior work experience in primary health care.

The finding that prior work experience in primary health care is associated with clinical competence development is interesting with regard to NP programmes that include clinical work experience as an entry requirement. According to the ICN [1], entry requirements for NP programmes in terms of work experience differ markedly between countries. For example, Gardner, Dunn, Carryer and Gardner [44] found that entry requirements across the 14 programmes they studied varied from zero to 5 years of experience. In the present authors’ previous study [16], we did not find evidence to recommend having work experience as an entry requirement. Due to the findings in the present study, that are significant yet minor, we remain inconclusive to a recommendation for having working experience as an entry requirement.

## Limitations

The PROFFNurseSAS II questionnaire has been evaluated for content validity [15] and reliability [16]. However, findings indicate that it may have issues related to its ability as a tool to assess the development of some aspects related to consultation, coaching and guidance, as well as collaboration at an advanced nursing level. This may be due to the fact that there are relatively few items in the questionnaire that pertain to these aspects, combined with a ceiling effect.

The value of self-assessment in nursing education is currently disputed, since it has been argued that nursing students at Bachelor’s level do not possess either sufficient faculty of self-reflection in relation to their actions, or the critical thinking skills necessary to carry out self-assessment [45]. The Dunning–Kruger effect has shown that poor performers overestimate their performance [46], thus raising doubts as to the construct validity of self-assessment approaches. However, Flynn, Valeberg, Tønnessen and Bing-Jonsson [47] found that Master’s students in nurse anaesthesia education significantly underestimated their clinical performance in relation to non-technical skills when compared with the assessments of their clinical supervisors. This finding supports the argument made in our previous study [16] that self-assessment is a valid and reliable approach to evaluations of APN education.

The present study does not attempt to evaluate the development of NPs’ clinical competence from the start to the end of their education, but from the start to the completion of their clinical courses in physical assessment, pathophysiology and advanced pharmacology, and their clinical studies. Before completing the NP Master’s programme, the students still have to complete their Master’s theses, which each offer 30 ECTS credits. Students are encouraged to choose topics with a clinical focus, or a project centred on professional development or quality improvement. Thus, since the students in this study had not yet fully completed their Master’s programme at the follow-up point, we suggest that more research is needed in order to investigate the students’ level of clinical competence as certified NPs.

We acknowledge that in general terms our sample size of 36 participants is limited in the context of a quantitative research project. However, the population of 46 students is also small. The study has achieved a high response rate and from the power analysis we have concluded that the sample size is appropriate. However, we recognise the problems associated with generalising our results on the basis of such a small sample size, and recommend further research using a larger sample.

## Conclusion

The greatest development in the NPs’ clinical competence observed in this study was in relation to direct clinical practice. This is in line with international standards and the intended learning outcomes of the NP Master’s programme. However, the results of the present study are inconclusive regarding the students’ development in the fields of consultation, coaching and guidance, and collaboration. Furthermore, we observed a lack of development in aspects concerning clinical leadership in the workplace. The students entered the NP programme with different levels of clinical competence in terms of direct clinical practice, but these differences were largely equalised during their education. Students with low clinical competence of direct clinical practice at baseline achieved significantly greater advances in their clinical competence during their education than those with high clinical competence at baseline. Clinical work experience in primary health care was a statistically significant, yet minor, predictor of the development of clinical competence among the students during the programme. The authors thus refrain from recommending that prior clinical experience should be an entry requirement for the NP programme. We believe that the results of the present study may be used to improve nurse practitioner education, and recommend that an evaluation of the current NP programme be carried out in order to determine whether the curriculum meets the academic standards of clinical leadership that are expected for advanced level nursing practitioners.

The Norwegian government has contributed by introducing an APN certification and regulations governing APN Master’s education in Norway. It is now imperative that the clinical sector works together with the NP students to integrate the NP role in the workplace. NP students must be given the opportunity to display and further advance their newly acquired clinical competence at advanced level so that potential health outcomes for patients can be measured. Further research into the development of NP students’ clinical competence should include studies of future NPs with clinical work experience in relevant and advanced fields of nursing practice in clinical settings. The objective here will be to evaluate the transfer of learning outcomes from education to clinical practice. With regard to the methodological dilemmas associated with self-assessment, we believe that it would be interesting to include NP students or NPs clinical preceptors in such studies in order to strengthen the validity of any findings.

## Data Availability

Due to the express wishes of the informants, the datasets used and analysed in the present study are not available. The corresponding author can be contacted.

## Abbreviations

APN: Advanced Practice Nursing
ICN: International Council of Nurses
OECD: Organisation for Economic Cooperation and Development
RNs: Registered Nurses
CNS: Clinical Nurse Specialist
NP: Nurse Practitioner
PHC: Primary Health Care
ECTS: European Credit Transfer System
PROFFNurseSAS II: The Professional Nurse Self-Assessment Scale of clinical core competencies II (survey questionnaire)
OSCA: Objective Structured Clinical Assessment
OSCE: Objective Structured Clinical Examination
